# Porphyria Cutanea Tarda in a Patient With Hereditary Hemochromatosis: A Complex Overlap Disorder

**DOI:** 10.7759/cureus.74091

**Published:** 2024-11-20

**Authors:** Bethani L DeMaria, Aaron J Franke

**Affiliations:** 1 Division of Hematology and Oncology, Sidney Health Center Cancer Care, Sidney, USA

**Keywords:** heme biosynthesis, hereditary hemochromatosis (hh), iron storage disorder, porphyria cutanea tarda, primary iron overload

## Abstract

We present a case of a 34-year-old woman with a 12-week history of blistering skin lesions, ultimately diagnosed with co-existing porphyria cutanea tarda (PCT) and hereditary hemochromatosis (HH) due to a homozygous C282Y *HFE* mutation. The patient's discovered genetic predisposition to iron overload played a key role in the development of clinically symptomatic PCT. Treatment with serial therapeutic phlebotomy was started, dramatically improving her symptomatic cutaneous disease, iron indices, and liver function tests. The case brings to the fore the need for thorough diagnostics including genetic testing and the early identification and treatment of iron overload in patients with PCT. This case emphasizes the clinical effectiveness of reducing plasma iron by phlebotomy and underscores the importance of intervention to prevent the long-term complications of pathologic iron overload in PCT. This case report serves to supplement the paucity of existing literature detailing the complex association between PCT and HH and the diagnostic challenges of identifying these commonly co-existing conditions.

## Introduction

Porphyria cutanea tarda (PCT) and hereditary hemochromatosis (HH) are distinct conditions that commonly co-exist in patients. The overlapping signs and symptoms associated with these disorders emphasize the importance of comprehensive diagnostics and the challenges faced in clinical practice. Porphyrias represent a group of disorders caused by enzymatic defects in the heme biosynthesis pathway. Porphyria cutanea tarda (PCT), the most common subtype of porphyria, is characterized by the deficient activity of uroporphyrinogen decarboxylase (UROD), leading to the accumulation of porphyrins resulting in photosensitive blistering skin lesions and liver dysfunction [1]. Hereditary hemochromatosis (HH) is an autosomal dominant disorder resulting from mutations in the *HFE* gene, causing excessive intestinal iron absorption and iron overload, leading to liver cirrhosis, cardiomyopathy, endocrinopathy, hypogonadism, and polyarthropathy [2]. The co-occurrence of PCT and HH is not uncommon due to overlapping pathogenic mechanisms of iron metabolism. Elevated hepatic iron stores exacerbate PCT by promoting oxidative damage to hepatocytes, further inhibiting UROD. Thus, the management of these patients requires an approach that addresses both iron overload and porphyrin accumulation. The clinical benefit of removing plasma iron through phlebotomy supports the prognostic role of iron overload in this disease. This case highlights the importance of the careful diagnostic consideration of co-existing *HFE* mutations and the early detection and treatment of iron overload in patients presenting with PCT.

## Case presentation

**Figure 1:**
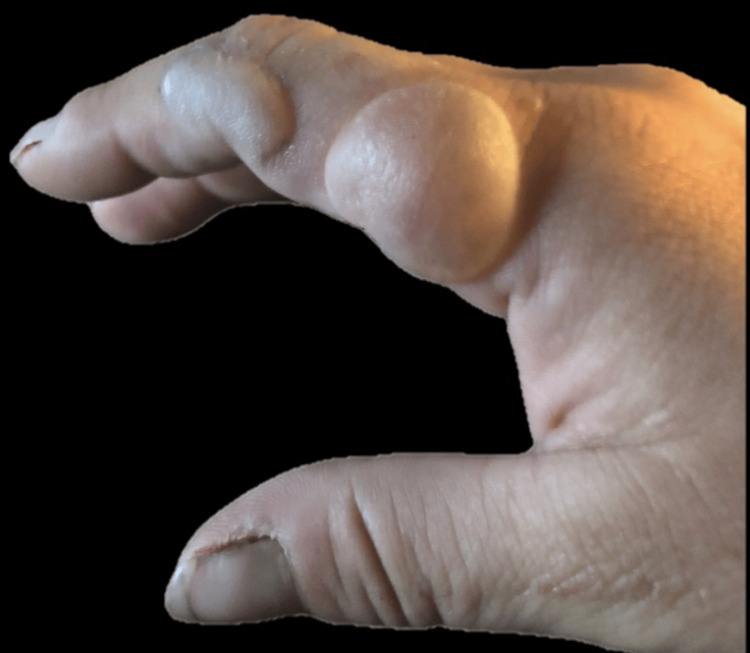
Bullous lesion on the hand of our patient with porphyria cutanea tarda

**Figure 2:**
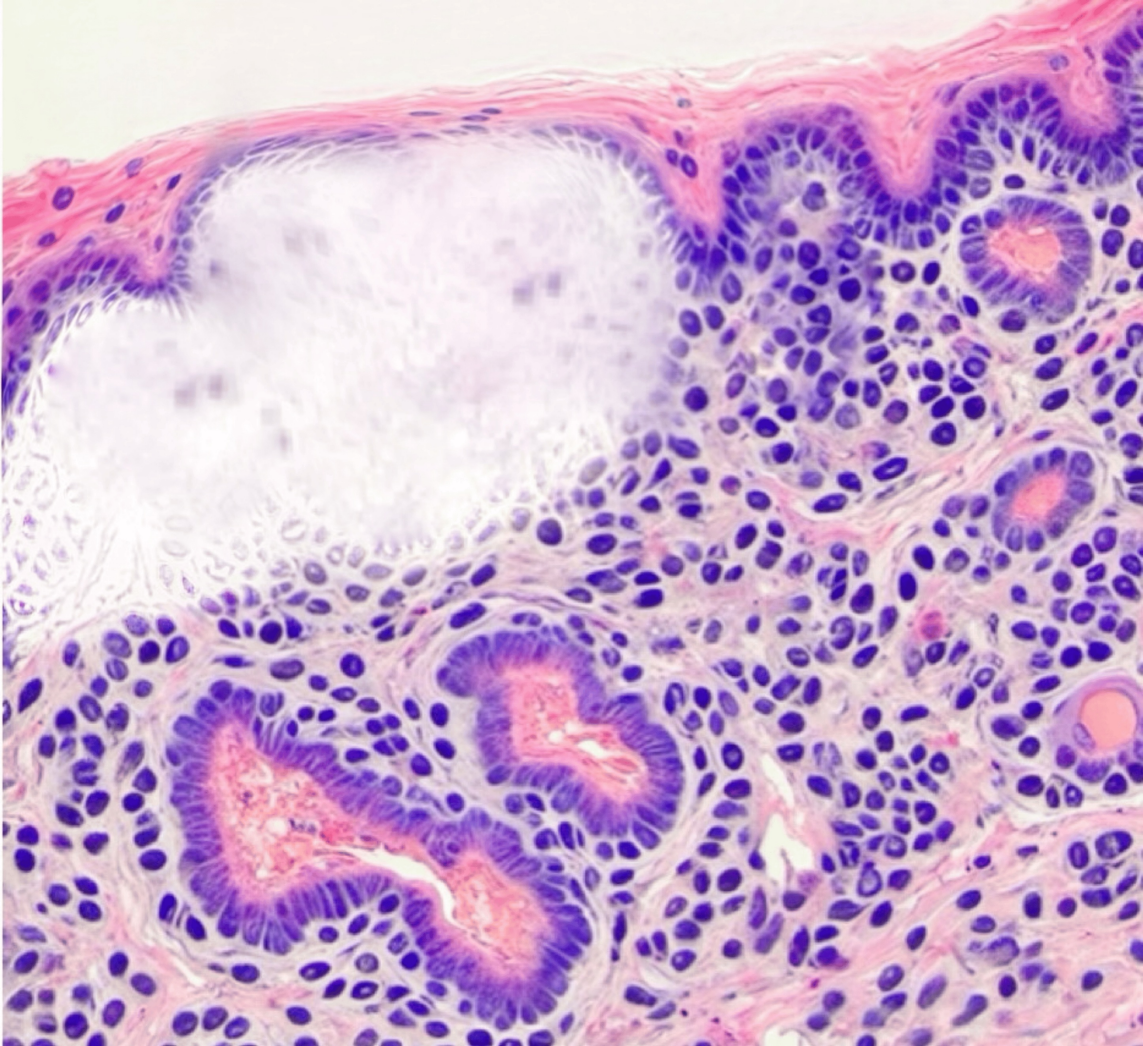
Histopathology: biopsy of a PCT bullous skin lesion The histopathologic examination of H&E and PAS ­staining demonstrates a pauci-inflammatory, cell-poor subepidermal bullae with the loss of the epidermis, the thickened basement membrane of capillary vessel walls, and perivascular PAS+ hyaline deposition within the dermis H&E, hematoxylin and eosin; PAS, periodic acid­-Schiff; PCT, porphyria cutanea tarda

The case describes a 34-year-old Caucasian woman with a 12-week history of a blistering skin disorder who was referred to hematology for further evaluation of suspected porphyria cutanea tarda (PCT). In midsummer, the patient presented with a rash and an eruption of numerous dorsal hand and finger erythematous, bullous skin lesions (Figure 1). She received multiple rounds of antibiotics and a three-week course of oral corticosteroids (prednisone 20 mg/day) from her local urgent care clinic, with minimal improvement. She was referred to dermatology and underwent a punch biopsy of the left index finger (dorsal proximal phalanx) and left dorsal hand. Figure 2 illustrates the histopathology from the biopsy, with classic PCT findings of pauci-inflammatory subepidermal blister and perivascular, amorphous hyaline deposits in the papillary dermis. Given the biopsy findings and strong clinical suspicion for a diagnosis of PCT, the consulting dermatologist initiated the patient on every-other-week therapeutic phlebotomy while awaiting referral to hematology.

Diagnostic workup

**Figure 3:**
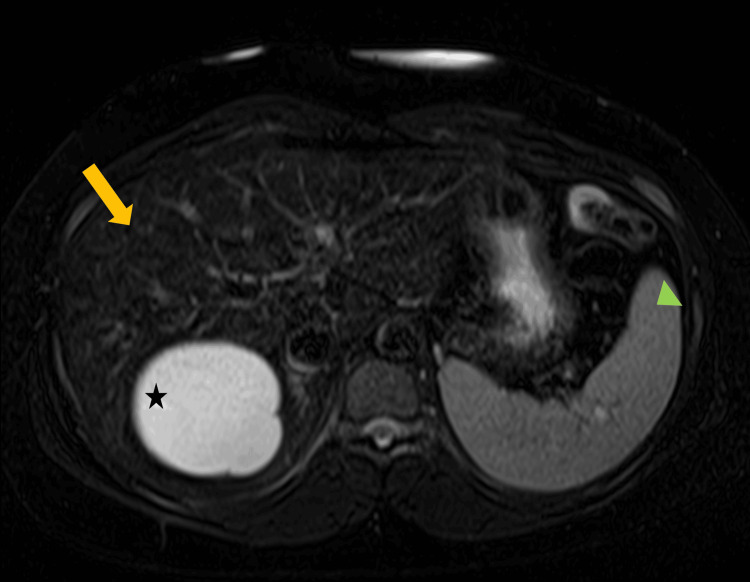
MRI liver protocol: radiographic hepatic siderosis Axial contrasted MRI (T2-weighted gradient echo) in a patient with iron overload secondary to PCT and HH. Contemporary MRI technology can accurately visualize and quantify excess hepatic iron. The visceral iron deposition causes a magnetic susceptibility artifact and T2 signal loss, resulting in decreased intensity on MRI (the darker the liver, the higher the iron content). Gradient in/out-phase and gradient echo MR sequences provide an important diagnostic clue of iron overload in patients presenting with PCT and elevated transaminases. In our patient, MRI demonstrates a typical finding in HH, with a sharp contrast between the dark liver (yellow arrow) and the "white" spleen (green arrowhead). The figure also shows an incidentally discovered 8.4 cm simple cyst in the upper pole of the right kidney (black star) MRI, magnetic resonance imaging; PCT, porphyria cutanea tarda; HH, hereditary hemochromatosis; MR, magnetic resonance

Results from the patient's pre-venesection workup are detailed in Tables 1-3. Other than transaminitis (hepatocellular pattern) and iron overload, the initial laboratory workup was unrevealing, with normal renal function, no endocrinopathy, white blood cell (WBC) count and differential within normal limits, negative viral hepatitis and human immunodeficiency virus (HIV) screen, and no hematologic abnormalities on peripheral blood smear. Abdominal magnetic resonance imaging (MRI) with and without intravenous contrast was performed, showing diffuse, heterogeneous hepatic reticulation and abnormal T1 signaling (Figure 3). *HFE* genetic testing using droplet digital polymerase chain reaction (ddPCR) detected biallelic (homozygous) C282Y variant mutations. Urine and plasma total porphyrin levels were both elevated (Tables 2, 3). The fractionation of urine and plasma porphyrins by mass spectrometry demonstrated a predominance of highly carboxylated porphyrins (uroporphyrin and hepta-, hexa-, and pentacarboxyl). Plasma aminolevulinic acid (ALA) and porphobilinogen (PBG) were normal, and only a mild elevation in urinary PBG was noted. Cardiac single-photon emission computed tomography (SPECT) imaging and liver biopsy were ordered but, due to socioeconomic and insurance issues, have not yet been performed. The results from a porphyria comprehensive genetic panel (next-generation sequencing) are pending.

**Table 1:** Laboratory values at diagnosis **HBV screen: hepatitis B surface antigen (HBsAg), hepatitis B surface antibody (anti-HBs), and hepatitis B core antibody, total (anti-HBc) WBC, white blood cells; MCV, mean corpuscular volume; RDW, red cell distribution width; Na, sodium; K, potassium; Cl, chloride; CO_2_, carbon dioxide; BUN, blood urea nitrogen; SCr, serum creatinine; eGFR, estimated glomerular filtration rate; TIBC, total iron-binding capacity; TSAT%, transferrin saturation; Alk Phos, alkaline phosphatase; ALT, alanine aminotransferase; AST, aspartate aminotransferase; T. Protein, total protein; T. Bili, total bilirubin; PT, prothrombin time; INR, international normalized ratio; aPTT, activated partial thromboplastin time; AFP, alpha-fetoprotein; HBV, hepatitis B virus; HCV Ab, hepatitis C virus antibody; HIV 1/2 Ag/Ab, human immunodeficiency virus type 1/2 antigen/antibody; HbA1C, hemoglobin A1c; TSH, thyroid-stimulating hormone; FT4, free thyroid hormone

Test Name	At Diagnosis	Reference Range
WBC (K/µL)	4.9	4-11
Hemoglobin (g/dL)	12	11.5-15.8
Hematocrit (%)	35	35-45
MCV (fL)	99.7	80-98
RDW (%)	12	11.5-15.5
Platelet Count (K/µL)	255	140-400
Na (mmol/L)	138	136-145
K (mmol/L)	4	3.5-5.1
Cl (mmol/L)	103	98-107
CO_2_ (mmol/L)	24.1	21-32
BUN (mg/dL)	13	7-18
SCr (mg/dL)	0.8	0.6-1
eGFR (mL/minute)	99	>60
Calcium (mg/dL)	8.7	8.5-10.1
Iron (µg/dL)	264	50-175
TIBC (µg/dL)	271	250-450
TSAT%	97.42	20-55
Ferritin (ng/mL)	2806	8-252
Vitamin B12 (рg/mL)	724	213-816
Alk Phos (U/L)	61	46-116
ALT (U/L)	151	14-63
AST (U/L)	125	15-37
T. Protein (g/dL)	7.6	6.4-8.2
Albumin (g/dL)	3.7	3.4-5
T. Bili (mg/dL)	1	0.2-1
PT (seconds)	10.4	9.3-11.4
INR	1.1	0.9-1.5
aPTT (seconds)	27	23-30
AFP (ng/mL)	2.3	0.0-8.8
HBV Screen**	Non-reactive	Non-reactive
HCV Ab Reflex	Non-reactive	Non-reactive
HIV 1/2 Ag/Ab	Non-reactive	Non-reactive
HbA1C (%)	4.3	<5.7
FT4 (ng/dL)	0.9	0.7-1.5
TSH (µIU/mL)	1.08	0.35-4.94

**Table 2:** Plasma porphyrins at diagnosis and post six-month phlebotomy TP: therapeutic phlebotomy

Plasma Porphyrins	At Diagnosis	Six-Month TP	References
Total Porphyrins (mcg/dL)	19.5	1.3	≤1.0
Uroporphyrin (mcg/g)	9.7	0.7	≤1.0
Heptacarboxyl porphyrin (mcg/g)	5.7	0.3	≤1.0
Hexacarboxyl porphyrin (mcg/g)	2.8	<0.1	≤1.0
Pentacarboxyl porphyrin (mcg/g)	0.8	<0.1	≤1.0
Coporphyrin (mcg/g)	0.2	<0.1	≤1.0
Protoporphyrin (mcg/dL)	0.2	<0.1	≤1.0

**Table 3:** Urine porphyrin laboratory results at diagnosis

Urine Porphyrins	At Diagnosis	References
Total Porphyrins (mcg/g)	6856.4	27-153.6
Uroporphyrin I (mcg/g)	8188.4	3.6-21.1
Uroporphyrin III (mcg/g)	1753.7	≤5.6
Heptacarboxyl porphyrin (mcg/g)	1556.2	≤3.4
Hexacarboxyl porphyrin (mcg/g)	10.2	≤6.3
Pentacarboxyl porphyrin (mcg/g)	168	≤4.1
Coporphyrin I (mcg/g)	159	6.5-33.2
Coporphyrin III (mcg/g)	20	4.8-88.6
Porphobilinogen (mmol/L)	2.1	≤1.3

Treatment and outcomes

The patient underwent serial therapeutic phlebotomy (450 mL) every two to four weeks, with a target ferritin of less than 50-100 ng/mL, aiming for low-normal to slightly iron-deficient range and a 33%-35% hematocrit hold parameter to limit symptomatic anemia. She significantly reduced her recreational alcohol intake and was provided education on proper sunlight avoidance and skin protection to mitigate exacerbating her photosensitive lesions. The addition of hydroxychloroquine for PCT treatment was discussed, but the patient was not interested. After six months of therapeutic phlebotomy, the patient had a marked improvement in pain, pruritus, and the appearance of all skin lesions; no new bullae appeared; and the patient continued to show healing/scarring of the previously erupted blisters. Her laboratory results at follow-up visits showed continued improvement in her iron indices and the normalization of liver function tests (Table 4). Total and fractionated plasma porphyrin levels were nearly all normalized (Table 2). Her follow-up urine sample to assess the response of urine porphyrins was contaminated by light and not performed. She has repeat plasma and urine porphyrin studies scheduled prior to her next visit. She remains on maintenance phlebotomy approximately every six to 12 weeks.

**Table 4:** Laboratory results at diagnosis and six months post phlebotomy TSAT%, percent iron saturation; Alk Phos, alkaline phosphatase; ALT, alanine aminotransferase; AST, aspartate aminotransferase; T. Bili, total bilirubin; TP, therapeutic phlebotomy

Test Name	At Diagnosis	Post Six-Month TP	References
TSAT%	97.42	38	20-55
Ferritin (ng/mL)	2806	113	8-252
Alk Phos (U/L)	61	64	46-116
ALT (U/L)	151	24	14-63
AST (U/L)	125	25	15-37
T. Bili (mg/dL)	1	0.5	0.2-1

## Discussion

**Figure 4:**
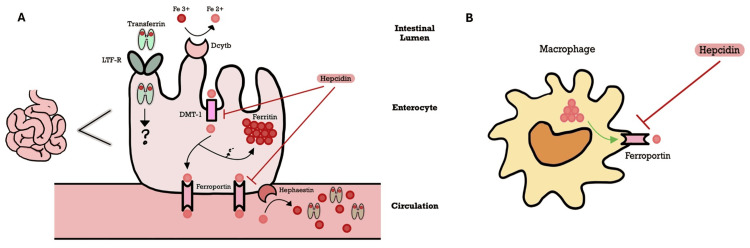
Iron transport: the hepcidin-ferroportin axis (A) Dietary iron (Fe) is primarily in ferric (Fe3+) form, requiring reduction to ferrous (Fe2+) heme using luminal ferrireductase duodenal cytochrome B (Dcytb) to facilitate absorption via transport across the enterocyte apical DMT1. Within the enterocyte, Fe2+ is stored as ferritin or exported across the basolateral membrane by FPN1. Hephaestin re-oxidizes iron back to ferric (Fe3+) form and is then bound to serum transferrin (Tf) prior to entering circulation. Increased levels of hepcidin promote the internalization and degradation of DMT1 and FPN1, decreasing both intestinal iron absorption and efflux into circulation [5]. (B) The recycling of iron through the consumption of senescent/damaged RBCs by specialized macrophages (e.g., splenic) is another primary contributor to iron homeostasis. During erythrophagocytosis, Fe is released within the macrophage, followed by cellular efflux via FPN1, which is ubiquitously expressed in all iron-metabolizing macrophages. The oxidized Fe3+ is then bound to circulating Tf, which serves as the major source of Fe for virtually all cells in the body. In PCT and HH, iron overload (ferritin, TSAT%) is sensed by hepatocytes, consequently increasing the production of hepcidin. Analogous to GI epithelial cells, hepcidin binds FPN1 on macrophages, triggering the internalization and degradation of the sole iron exporter [6]. All figures presented in this study are original works created by the authors and have not been previously published elsewhere DMT1, divalent metal transporter 1; FPN1, ferroportin-1; GI, gastrointestinal; RBCs, red blood cells; TSAT%, transferrin saturation; PCT, porphyria cutanea tarda; HH, hereditary hemochromatosis; LTF-R, lactotransferrin receptor

*HFE* gene mutations causing aberrant iron metabolism are the pathophysiologic hallmark of hereditary hemochromatosis (HH) [3]. *HFE* encodes a crucial component in the iron-sensing protein complex on the surface of hepatocytes [1]. Figure 4A, 4B illustrates a model of the key modulator of hepatic iron homeostasis, the "hepcidin-ferroportin axis." Hepcidin, primarily synthesized in the liver, binds to ferroportin, a transporter on the basolateral membrane of enterocytes, to induce endocytosis and degradation. In this way, elevated hepcidin levels negatively regulate ferroportin, inhibiting the absorption of dietary iron in the gastrointestinal (GI) tract, and prevent the release of iron from recycled hepatocytes and macrophages [4].

**Figure 5:**
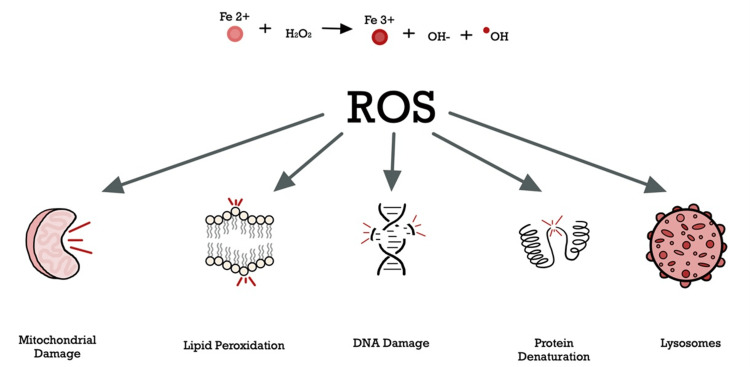
Iron-mediated oxidative stress: the cellular response Once plasma iron concentrations exceed transferrin-binding capacity, excess non-transferrin-bound iron (NTBI) is taken up by cells throughout the body. NTBI can shift between two redox states, and this high reactivity triggers the intracellular formation of reactive oxygen species (ROS) through the Fenton reaction. The cellular consequences of excessive ROS production have been demonstrated to play a pivotal role in the pathogenesis of several end-organ failure diseases (e.g., cirrhosis and cardiomyopathy), which remain a major cause of morbidity and mortality in patients with HH [9] HH: hereditary hemochromatosis

In HH, the defective *HFE *complex leads to the decreased production of hepcidin, tipping the axis toward an unchecked ferroportin, resulting in an inappropriately increased iron absorption and efflux, despite high levels of transferrin (Tf)-bound iron in plasma [7]. The high serum transferrin saturation (>60%) allows the abundance of non-transferrin-bound circulating iron to accumulate in various organs (e.g., the liver, pancreas, heart, and endocrine glands). This intracellular iron buildup generates reactive oxidative stress (Figure 5), DNA damage, cellular necrosis, and fibrosis, underscoring the vital consequences of chronic iron overload, begetting conditions such as cirrhosis, cardiomyopathy, and pancreatic insufficiency [8].

**Figure 6:**
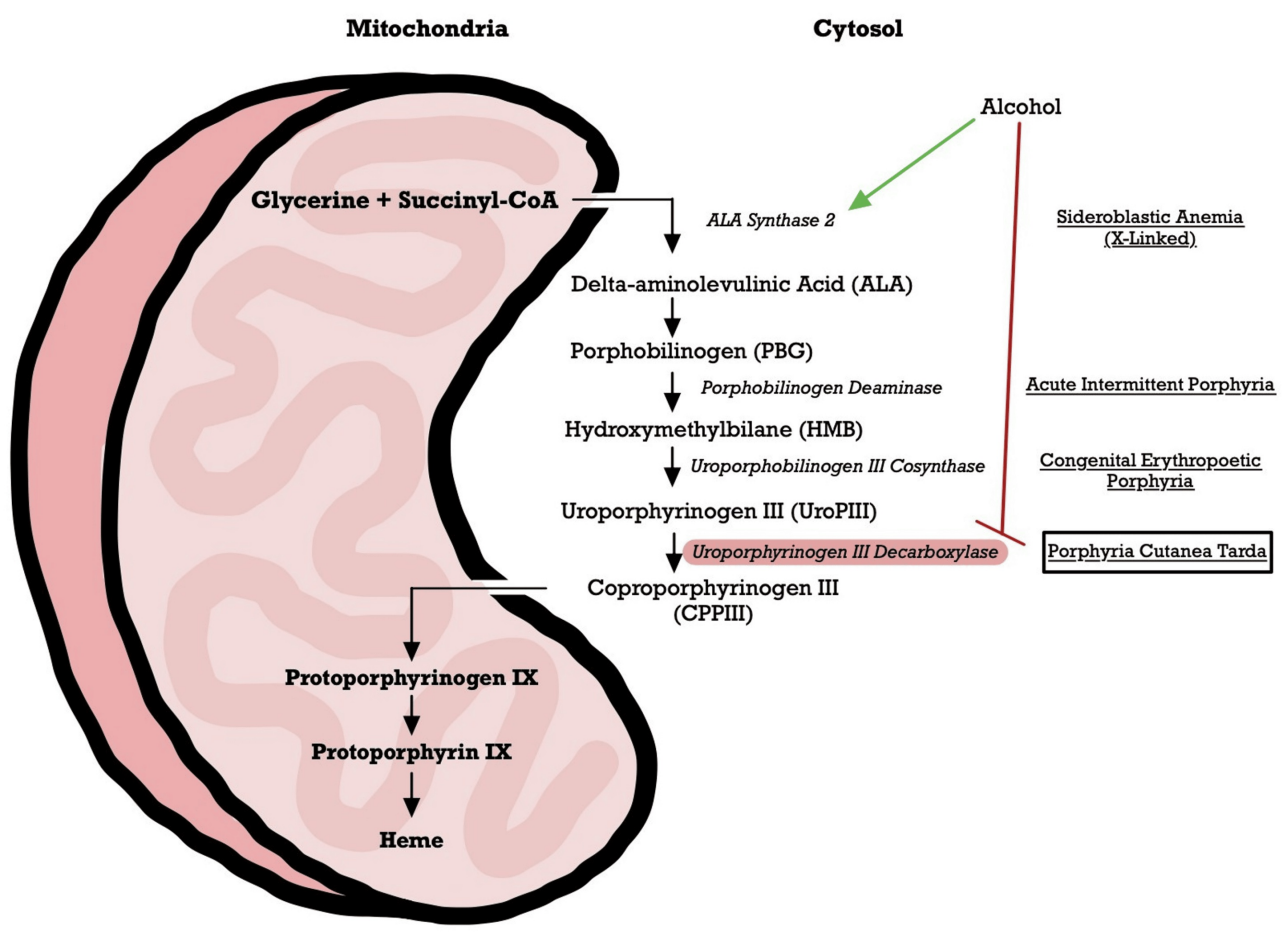
The heme biosynthetic pathway Heme synthesis begins in the mitochondria with the formation of delta (δ)-aminolevulinic acid (ALA) from glycine and succinyl-CoA via ALA synthase (ALAS), the rate-limiting step in the liver. δ-ALA subsequently undergoes six additional enzymatic reactions to yield protoporphyrin, which is then coupled to iron to form heme. Uroporphyrinogen decarboxylase (UROD) is the fifth enzyme in the heme synthesis pathway, catalyzing the four-step decarboxylation of uroporphyrinogen III and I to coproporphyrinogen III and I. Hepatic UROD is inhibited in all patients with PCT (only ~20% harbor an inherited UROD pathogenic mutation). In PCT, alcohol consumption exacerbates the disease further by increasing iron absorption and free radical production, stimulating hepatic δ-ALA, inhibiting uroporphyrinogen III decarboxylase, in addition to being independently hepatotoxic [11] CoA, coenzyme A; PCT, porphyria cutanea tarda

Porphyrias are heterogeneous disorders resulting from enzymatic defects in heme biosynthesis, as detailed in Figure 6. Porphyria cutanea tarda (PCT) is the most common nonacute (cutaneous) porphyria, caused by deficient uroporphyrinogen decarboxylase (UROD) in hepatocytes [10]. Uroporphyrinogen decarboxylase catalyzes the fifth enzymatic step in heme biosynthesis, and reduced UROD activity causes the hepatic and cutaneous accumulation of porphyrinogens. These highly carboxylated substrates are then oxidized by sunlight to photosensitize porphyrins, activating an inflammatory cascade with blistering lesions and hyperpigmentation on sun-exposed skin.

PCT is classified as sporadic (type 1) or familial (type 2). In sporadic PCT (80% of cases), the patients do not harbor UROD mutations, and symptoms do not typically manifest until the enzymatic activity falls below 20% of normal [12]. Patients with familial PCT (20% of cases) inherit a heterozygous UROD pathogenic variant, but due to incomplete penetrance and 50% retained enzyme activity, the majority (>60%) never develop symptoms unless provoked by external factors (alcohol, viral hepatitis, HIV, oral estrogen, and iron overload). In both sporadic and familial PCT, studies demonstrate reduced UROD catalytic activity despite normal UROD protein levels, suggesting the generation of a UROD inhibitor that is highly dependent on the presence of excess hepatic iron [1,13]. Serial phlebotomy to remove excess iron resolves most PCT-related symptoms [14].

**Figure 7:**
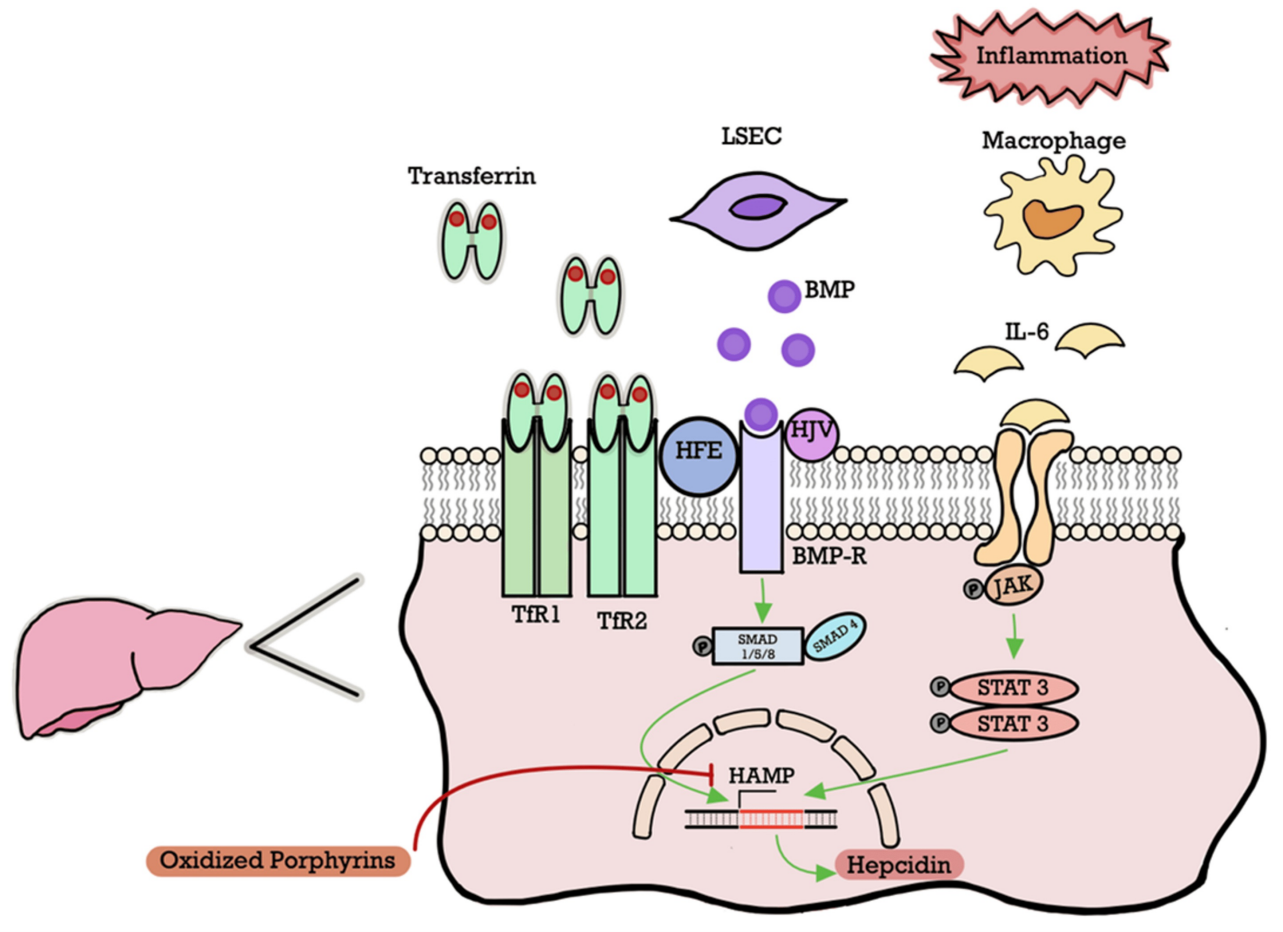
Iron-dependent hepatic regulation of hepcidin Hepcidin production is transcriptionally regulated in proportion to plasma and tissue iron levels. In conditions of overload (HH and PCT), plasma iron concentrations exceed the iron-binding capacity of transferrin, and the excess non-transferrin-bound iron is taken up by hepatic endothelial cells and upregulates the expression of hepcidin to mitigate any further iron absorption, maintaining homeostasis. Extracellular iron-sensing (holo-transferrin sensing) is dependent on TFR1, TFR2, and *HFE*, on the surface of hepatocytes. Bone morphogenetic proteins (BMP) produced by liver sinusoidal endothelial cells (LSECs) bind to several key hepcidin regulatory molecules (BMP receptors, *HFE*, and HJV) to form a transmembrane complex. This activates downstream SMAD signaling, ultimately increasing hepcidin transcription through the *HAMP* promoter. Inflammatory states enhance hepcidin production through IL-6-mediated JAK/STAT signaling, working synergistically with the BMP pathway [5,16] HJV, hemojuvelin; IL-6, interleukin 6; JAK, Janus kinase; SMAD, small mother against decapentaplegic; STAT, signal transducer and activator of transcription; TFR1/2, transferrin receptor 1/2; HH, hereditary hemochromatosis; PCT, porphyria cutanea tarda

The prevalence of *HFE* variants is approximately 2%-27% in PCT patients (23% heterozygous and 19% homozygous for C282Y and 23% heterozygous and 8% homozygous for H63D) [15]. Hepcidin production is also impaired in PCT patients without *HFE* mutations, suggesting that other factors reduce its expression (Figure 7). The complex role of iron in the pathogenesis of hepatic siderosis in PCT is incompletely understood, but data exists to support a hypothesis of an iron-mediated pathway of direct UROD inhibition and through indirect suppression as a vital cofactor in generating the UROD inhibitor [2,11]. The hepatic accumulation of oxidized porphyrins also decreases the expression of *HAMP*, the gene encoding hepcidin, increasing iron export in enterocytes and macrophages and creating a pathogenic feedback loop that continuously generates the UROD inhibitor responsible for manifestations of PCT [4].

Both HH and PCT demonstrate variable penetrance, suggesting that phenotypic expression is greatly influenced by HH-specific genotypes and environmental factors [2]. HH typically manifests in middle age, with a delayed presentation in women due to menstrual iron loss. The nonspecific nature of HH symptoms often delays diagnosis, increasing the risk of irreversible end-organ damage. However, when *HFE* mutations coincide with environmental triggers, PCT can manifest earlier, as demonstrated in our patient where alcohol use and iron overload precipitated early PCT [12].

It is important for clinicians to recognize the potential for cognitive errors to avoid diagnostic (anchoring) biases in these overlap disorders, as concurrent PCT/HH portends a poorer prognosis than PCT alone, primarily due to advanced liver disease [17,18]. Therapeutic phlebotomy effectively addresses both conditions by removing excess iron, mobilizing porphyrins, and enhancing hepatic UROD activity [7]. Our patient's case exemplifies a successful treatment response, achieving the normalization of laboratory parameters and the near-complete resolution of her blistering skin disease within six months through serial phlebotomy and alcohol cessation.

## Conclusions

This case highlights the clinical presentation and management of porphyria cutanea tarda (PCT) in a 34-year-old woman with previously undiagnosed hereditary hemochromatosis (HH) due to homozygous C282Y *HFE* mutation. The combination of alcohol use and an underlying genetic predisposition precipitated PCT development in this otherwise healthy patient. Her characteristic blistering skin lesions prompted a thorough evaluation, leading to the early detection of iron overload and the initiation of therapeutic phlebotomy. This report emphasizes that PCT can serve as an early indicator of underlying HH, and rapid diagnosis enables timely intervention to prevent long-term sequelae associated with chronic iron overload. Our case contributes to the existing literature to reinforce the importance of comprehensive evaluation for co-occurring HH in patients presenting with PCT, particularly in those with contributing risk factors.
